# Menstrual cycle modulates the effect of BDNF Val66Met variant on category learning

**DOI:** 10.1186/s13293-026-00856-2

**Published:** 2026-03-01

**Authors:** Mateja Perović, Jianqi Hou, Michael L. Mack

**Affiliations:** Department of Psychology, University of Toronto, Toronto, ON M5S 3G3 Canada

**Keywords:** Behavioral genetics, Learning and memory, Concept formation, Estrogen, Brain-derived neurotrophic factor

## Abstract

**Background:**

Both brain-derived neurotrophic factor (BDNF) and ovarian hormones are powerful neuromodulators, yet evidence of their impact on human cognition remains mixed. As prior work has studied them in isolation, examining their interacting effects presents a key empirical opportunity for explicating their effects on cognition.

**Methods:**

We genotyped participants for the BDNF Val66Met single nucleotide polymorphism, which is associated with less efficient activity-dependent BDNF secretion and altered hippocampal function, and examined their performance on a complex learning task at two points in the menstrual cycle: early follicular (characterized by low levels of ovarian hormones) and late follicular (characterized by high estradiol).

**Results:**

While *met* carriers showed advantages during the early follicular timepoint, *val* homozygotes outperformed them at the late follicular timepoint. Furthermore, effects in *met* carriers were largely driven by increased sensitivity to both absolute levels and changes in levels of estradiol.

**Conclusions:**

The current findings provide the first evidence of BDNF Val66Met interacting with the menstrual cycle to predict cognition, demonstrate nuanced genotype- and hormone-specific outcomes, and underscore the importance of studying effects of interacting biological systems on human cognition.

**Supplementary Information:**

The online version contains supplementary material available at 10.1186/s13293-026-00856-2.

## Highlights

- Effects of BDNF genotype on human cognition vary by point in menstrual cycle.
- Met allele carriers outperform val homozygotes in the early but not late follicular timepoint.
- Met carriers show increased sensitivity to absolute levels of estradiol and variation in estradiol levels.
- Higher magnitude of change in estradiol between timepoints is associated with cognitive performance decrements in met carriers and improvements in val homozygotes.
- BDNF genotype may affect hormonal sensitivity in healthy populations.

**Supplementary Information:**

The online version contains supplementary material available at 10.1186/s13293-026-00856-2.

## Abstract (plain language summary)

Both brain-derived neurotrophic factor (BDNF), a protein that affects neuronal growth and survival, and ovarian hormones have established effects on cognition. However, past studies have largely studied their effects in isolation even though BDNF and hormones interact at a molecular level. For example, the ovarian hormone estradiol can boost BDNF levels. This study uses BDNF genotype as a way of determining baseline BDNF availability and the menstrual cycle as a natural manipulation of ovarian hormone levels. Participants completed a complex cognitive task at two points in the menstrual cycle: one typically characterized by low ovarian hormone levels, and one typically characterized by high levels of estradiol. Results indicated that menstrual cycle effects on cognition differed by BDNF genotype. This is interesting for several reasons. Firstly, this is the first study to demonstrate an interaction between BDNF genotype and the menstrual cycle when it comes to cognition. This is especially important given that prior research suggested that both BDNF genotype and the menstrual cycle were important for cognition, but previous studies examining each of those factors in isolation have yielded mixed results. This work suggests that we can learn more about cognition if we examine these interacting biological systems together.

**Supplementary Information:**

The online version contains supplementary material available at 10.1186/s13293-026-00856-2.

## Background

While ovarian hormones have been traditionally overlooked in neuroscience research, a large body of work developed over the recent decades now demonstrates that they have significant and widespread effects on brain and behavior, across species, in both health and disease [1–9]. However, significantly less is known about individual differences in the effects of these hormones on cognition despite both cognitive [2, 4, 10] and clinical [11–13] literatures suggesting significant variance in the extent human behavior is affected by hormonal, and associated neural, changes across the menstrual cycle. This gap has major implications, not only for our ability to develop a complete account of human cognition, but also for understanding risk factors for and trajectories of psychiatric and neurological disorders with known sex differences in prevalence or presentation [6, 7, 14, 15].

The literature on behavioral effects of genetic variants on human cognition in health and disease has similarly shown rich but mixed results, with effects largely varying by domain and population examined [16–22]. Because ovarian hormones are powerful neuromodulators [8, 23–25] known to affect gene expression [6, 26, 27], it is likely that at least some of the controversies present in the literatures on the effects of hormone and genetic variants on cognition could be resolved by examining the interacting effects of these factors.

Brain derived neurotrophic factor (BDNF), which promotes proliferation of neurons and synapses [28] in a range of brain areas that support core cognitive functions [29] is of particular interest as its expression is significantly modulated by ovarian hormone estradiol [30]. BDNF is most abundantly expressed in the hippocampus [31], where it supports synaptic plasticity and long-term potentiation as revealed by rodent models [32–34]. A common single-nucleotide polymorphism in the BDNF gene (Val66Met) is associated with alterations in BDNF secretion. Carriers of the *met* allele have reduced activity-dependent BDNF secretion and less successful BDNF intracellular trafficking than individuals homozygous for the *val* allele [35]. Most early human studies have suggested that this lower availability results in worse episodic memory performance in younger and middle-aged adults [35–37] as well as smaller hippocampal volume (for a meta-analysis across the adult lifespan see: [38]). However, the universality of these effects has been called into question in recent reviews and meta-analyses [17, 22, 39].

Critically, estradiol increases BDNF levels in the hippocampus [30, 40], augments BDNF release at the dentate gyrus (DG) and cornu ammonis 3 (CA3) hippocampal subfields [41], and predicts BDNF expression across both the human menstrual cycle and its animal analogues [42–44]. Furthermore, BDNF Val66Met polymorphism interacts with estradiol to predict hippocampal-dependent cognition. In rodents, performance of low-BDNF, *met* carriers improves with higher estradiol whereas the opposite is true for high-BDNF, *val* carriers [45]. In humans, hippocampal function in naturally cycling women may be modulated by estradiol in a genotypically specific manner. Specifically, women who carry the *met* allele show increased hippocampal activation during a working memory task in the presence of pharmacologically increased estradiol levels [46], further demonstrating the interactive relationship between estradiol and BDNF genotype. Although limited by a lack of control for female hormonal status at the time of testing, sex differences in the effects of BDNF genotype on human cognition in cohorts spanning the typical female reproductive lifespan (ages 18–50 [47, 48]) may also be suggestive of underlying hormonal effects.

However, despite reported sex differences in the effects of BDNF Val66Met on cognition [47–51] and strong evidence of interactive effects of estradiol and BDNF in the brain offered by animal studies [41, 45, 52], research on ovarian hormones, BDNF Val66Met, and human cognition is largely lacking. The menstrual cycle presents a useful model for studying effects of ovarian hormones, as hormonal changes across the cycle form distinct endocrine profiles which act as natural conditions researchers can use to tease apart effects of different ovarian hormones on cognition. Yet, no studies to date have leveraged this empirical opportunity to study interactions between BDNF Val66Met and the menstrual cycle in predicting cognition.

Here, we aim to examine hormone-gene effects on human cognition at two points in the menstrual cycle, during the early follicular (EF; characterized by low hormone levels) and late follicular (LF; characterized by high estradiol) periods. Our choice to focus on the follicular phase is informed by the findings of a prior cross-sectional study [59] which found significant variation in the cognitive measure used in the current study within the first half of the menstrual cycle. The cognitive measure we use, category learning, not only requires careful coordination of multiple core cognitive processes (memory, learning and attention) but it has also been distinctly associated with function of the hippocampus [53–59], the brain region where BDNF is most abundant [31] and where effects of estradiol have been repeatedly demonstrated through a range of neuroimaging modalities [4, 60, 61]. We utilize the type of categorization most distinctly associated with hippocampal function: rule-plus-exception (RPE) category learning. This type of category structure requires both general categorization governed by simple rules, as well as more sophisticated encoding of rare exception items which defy initially learned rules due to their high visual similarity to the opposite category. Successful exception categorization necessitates recruitment of hippocampal pattern separation, completion, and associative memory processes [54, 55, 57].

We expect to see effects of menstrual cycle phase on categorization of these exceptional category items, as both exception categorization and the related process of pattern separation have previously been shown to vary across the menstrual cycle [62, 63]. Furthermore, we expect to find interactive effects of menstrual cycle session and BDNF Val66Met on task performance, with exception categorization varying across the sessions in a genotype-specific manner.

## Methods

### Participants

Participants (age range: 18–30) were recruited via social media (Meta, Instagram) ads and asked to complete a brief eligibility survey. The eligibility survey was followed up with an in-depth phone interview for final eligibility determination. Participants were invited to take part in the study if they fulfilled the following criteria: not pregnant or breastfeeding, not using hormonal contraceptives – at present or in the past 6 months, reporting regular menstrual periods, menstrual cycles within the 21–35 day range, tracking menstrual cycles using an app or a calendar, no history of neurological conditions such as seizures or traumatic brain injury with loss of consciousness, normal or corrected-to-normal vision, normal or corrected-to-normal hearing, and English fluency. Our target sample size was 58, based on a G*Power [64, 65] analysis for a 2 × 2 repeated measures design, within-between interactions with an effect size of Cohen’s *f* = 0.45 and a significance level of 0.05. We based this effect size on previous studies of visual memory across the menstrual cycle [66] and working memory as a function of menstrual cycle and a genetic factor associated with working memory [67].

### Procedure

Participants completed two testing sessions. A rule-plus-exception category learning task was administered, followed by a test of general cognitive ability, and a demographic questionnaire including questions related to their menstrual cycle. Participants then provided two saliva samples - one to be analyzed for estradiol and progesterone, and one to be genotyped for BDNF Val66Met. Each of the tasks participants completed had two versions to avoid learning effects between the sessions. Task versions, as well as timing of the first session (early vs. late follicular) were counter-balanced across participants. Participants received monetary compensation for their participation in the study. All research protocols used in the study were approved by the University of Toronto Research Ethics Board. All experimental procedures were conducted in accordance with the University of Toronto Research Ethics Board and all participants provided written informed consent.

### Task

**Fig. 1:**
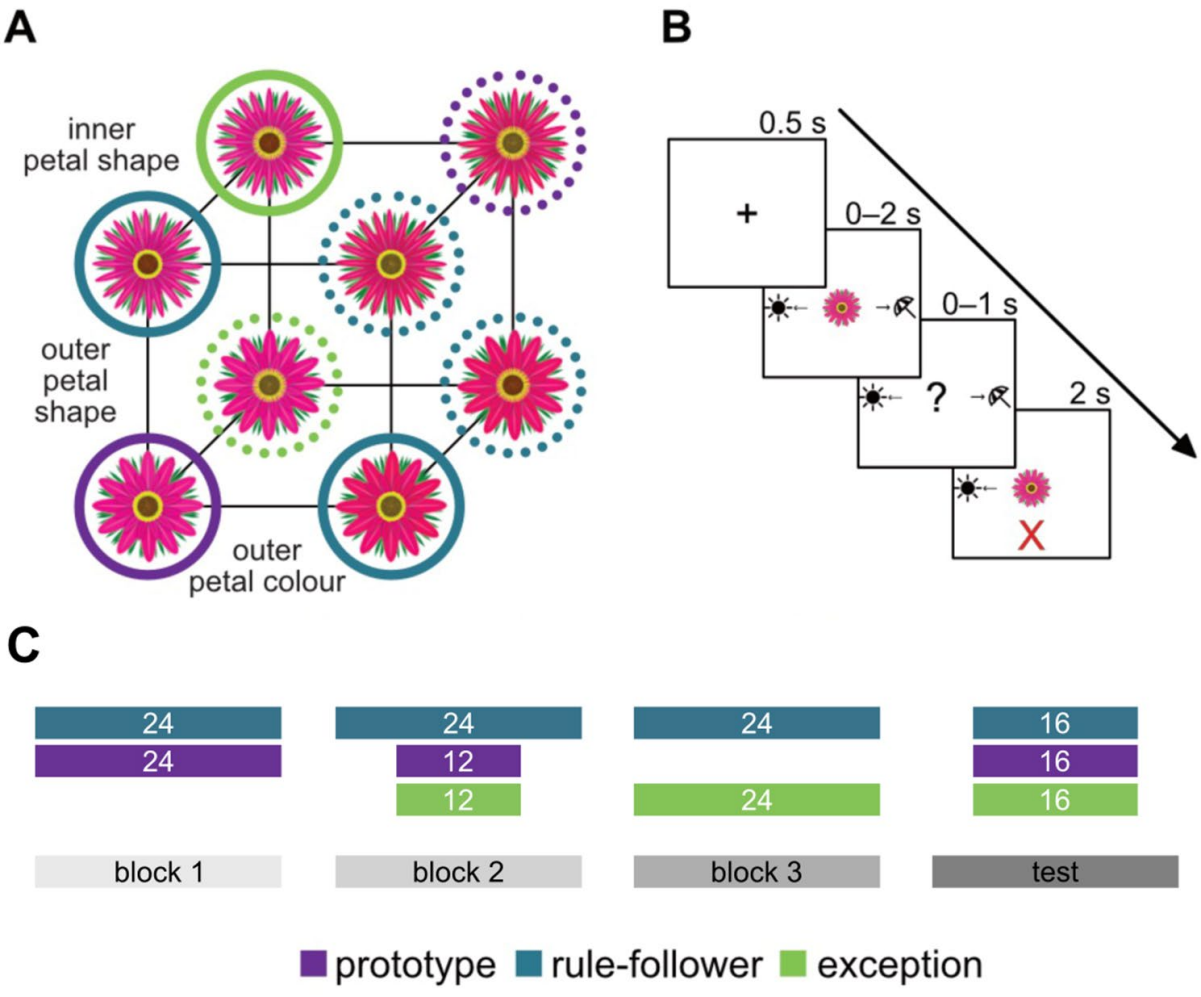
Category structure and experimental trial schematic (note: there were two, counterbalanced, stimuli sets, with alternate set using birds instead of flowers). (**A**) Stimuli consisted of three binary-valued dimensions with categories defined by a rule-plus-exception structure (solid and dotted circles denote category membership). Stimuli were either prototypes (purple; maximally dissimilar across categories), rule-followers (blue; more similar to their category prototype than to the prototype of the opposite category), or exceptions (green; more similar to the prototype of the opposite category). (**B**) Stimuli had to be categorized into one of two categories (“prefers shade” vs. “prefers sun”). Learning trials (3 in total) consisted of a fixation period (0.5s), presentation of the flower stimulus (2s), a response window (1s), and corrective feedback (2s). The task ended with a no-feedback test block. (**C**) The first learning block only included prototype and rule-follower items for initial exposure to simple category rules. Exceptions were introduced in the second learning block as their later introduction improves performance [54]. Final learning of all stimulus types was assessed in the test block

Participants completed a RPE categorization task [54, 68] consisting of three learning blocks and a no-feedback test block. Throughout the experiment, participants viewed 10 images of either flowers or birds, depending on the task version, with three binary-valued diagnostic dimensions and one non-diagnostic dimension. Stimuli had to be categorized into one of two categories (“prefers shade” vs. “prefers sun” for flowers; “prefers cold climate” vs. “prefers warm climate” for birds). More detailed descriptions of the task are provided in prior published work [54, 62] and Fig. 1.

### Control measures

To control for potential baseline individual differences in cognitive ability, participants completed Integrative Cognitive Assessment [69], a 5-minute rapid categorization task measuring speed and accuracy of processing visual information as a proxy for general cognitive ability. This is a test of rapid information processing across a network of cognitive mechanisms including visual perception, retrieval of and comparison to semantic knowledge, and motor response. It has been validated across adult age groups [69]. Participants were shown a series of rapidly flashing images and asked to indicate whether an image contained an animal or not by pressing on the left and right keyboard arrows. Each image was presented for 100 ms followed by a 20 ms inter-stimulus interval, and a 250 ms mask. Additionally, we administered the Perceived Stress Scale [70] and the Center for Epidemiologic Studies Depression Scale [71] to control for any cycle-related changes in stress or mood.

### Menstrual cycle timing

Participants reported their average cycle length as well as the current day of their cycle (assuming the first day of their menstrual period was day 1) in an initial phone screening. When reporting current day of their cycle and average cycle length, participants were required to consult their tracker app or calendar for a more accurate report (85% of participants used a tracker app). For participants using a calendar (without automated calculation of cycle length), the previous four cycles were used for calculation of the average cycle length. Cycle information was used to book testing sessions, so that the first session occurred 2–5 days and the second session 10–15 days after menses onset. Participants were asked to let the research team know if anything about their cycle changed (e.g., menses was delayed or started sooner than expected). This request was made during the initial phone screening and repeated in reminder emails sent three days and one day before their early follicular study session. Participants confirmed both their attendance and current day of their cycle leading up to the study sessions. This ensured that participants were tested in the expected range of the cycle.

### Hormone data

Two saliva samples (approximately 2 mL per sample) were collected to assess salivary estradiol and progesterone, one at each study session, with the aim to keep timing of saliva collection consistent between sessions for each participant (median time difference between the sessions was one hour). Participants were instructed to abstain from food for at least one hour prior to the session and from water or chewing gum for at least 30 min before saliva collection. Samples were collected using the passive drool method guided with sterilized straws. Samples were frozen immediately upon collection at − 20 °C. All samples were assayed at the Salimetrics’ SalivaLab (Carlsbad, CA) using the Salimetrics Salivary Progesterone Assay Kit (Cat. No. 1–1502), without modifications to the manufacturers’ protocol. Samples were thawed to room temperature, vortexed, and then centrifuged for 15 min at approximately 3,500 RPM (1,500 x g) immediately before performing the assay. Samples were tested using a high sensitivity enzyme immunoassay (Cat. No. 1–3702 for estradiol and Cat. No. 1–1502 for progesterone). Sample testing was performed in duplicate.

Sample test volume was 100 µl of saliva per determination for estradiol, and 50 µl of saliva per determination for progesterone. For estradiol, the assay had a lower limit of sensitivity of 0.1 pg/mL, a standard curve range from 1 to 32 pg/mL, an average intra-assay coefficient of variation of 7.13%, and an average inter-assay coefficient of variation of 7.45%. For progesterone, the assay had a lower limit of sensitivity of 5 pg/mL, a standard curve range from 10 to 2430 pg/mL, an average intra-assay coefficient of variation of 6.20%, and an average inter-assay coefficient of variation of 7.55%. The sensitivity for both hormones met the manufacturers’ criteria for accuracy and repeatability in Salivary Bioscience and exceeded the applicable NIH guidelines for Enhancing Reproducibility through Rigor and Transparency.

### Genotype data

Saliva samples were collected using Genotek Oragene saliva collection kits and stored at room temperature until analysis. Genotyping for the BDNF Val66Met polymorphism was performed at The Centre for Applied Genomics, The Hospital for Sick Children, Toronto, Canada using pre-designed TaqMan^®^ SNP Genotyping Assays (C__25746809_50 and C__11592758_10, Life Technologies Inc., Carlsbad, CA, USA). The 10 ul reaction mix consisted of 5 ul Accustart Genotyping Toughmix Low Rox (QuantaBio, Beverly, MA, USA), 0.25 ul of 40X combined primer and probe mix, 2.75 ul water and 50–100 ng of DNA template. Cycling conditions for the reaction were 95 C for 10 min, followed by 40 cycles of 94 C for 15 s and 60 C for 1 min. Samples were analyzed using the ViiA™ 7 Real-Time PCR System and analyzed using ViiA™7 software. All samples yielded clear genotype calls with no ambiguous clustering. Observed genotype frequencies did not deviate from Hardy-Weinberg equilibrium (*χ*^*2*^ = 2.99 [1], *p* = 0.08).

### Statistical analysis

All statistical analyses were conducted in R (version 4.1.1), and significance level was set at α = 0.05. We examined learning performance as a function of stimuli type, BDNF Val66Met and menstrual cycle session with linear mixed models that included random intercept for participants. *Met* carriers were combined into one group (*met/met* + *val/met*) and compared to *val* homozygotes, mirroring the most common approach in research on BDNF genotype and cognition [72–76]. Hormone effect analyses also utilized linear mixed models that included random intercept for participants, and learning performance was modeled as a function of hormone levels, BDNF genotype, and cycle phase. Learning performance in the late follicular timepoint was also modeled as a function of the magnitude of change in hormone levels between the early and late follicular timepoints and BDNF genotype using general using linear models. Confidence intervals (95%) were calculated for all analyses. Sensitivity analyses controlling for age and education, and controlling for any medication reported at time of testing are provided in the supplement.

## Results

Sixty-four participants took part in the study, with 60 completing both sessions (age: 27.2 ± 4.7; years of education: 17.5 ± 2.5). Average menstrual cycle length was 29.2 ± 4, with the early follicular timepoint occurring on cycle day 3.2 ± 1.3 and the late follicular timepoint on day 12 ± 1.4. All participants tracked their cycles using an app or a calendar. The BDNF allele distribution was as follows: 60% *val/val*, 40% *met* carrier. Most participants (40%) reported ethnicity of East or Southeast Asian origin, followed by South Asian origin (20%), European origin (16.7%), West Central Asian and Middle Eastern origin (6.7%), multiple ethnic origins (5%), Latin, Central and South American origin (5%), Caribbean origin (3.3%), African origin (1.7%), and other ethnic origin (1.7%).

There were no between-session differences in terms of perceived stress, depression or overall cognitive ability (both *p* < 0.05). There were no differences in progesterone levels between the two sessions. This was expected as progesterone remains low throughout the follicular phase. We also saw no difference in estradiol levels between the sessions, likely due to high natural variance in ovarian hormone levels across the menstrual cycle [77]. Consequently, estradiol and progesterone were controlled for in initial analyses predicting task performance by timepoint and a subset of participants with typical hormonal changes was analyzed in analyses directly examining hormonal effects on task performance.

### Task performance

**Fig. 2:**
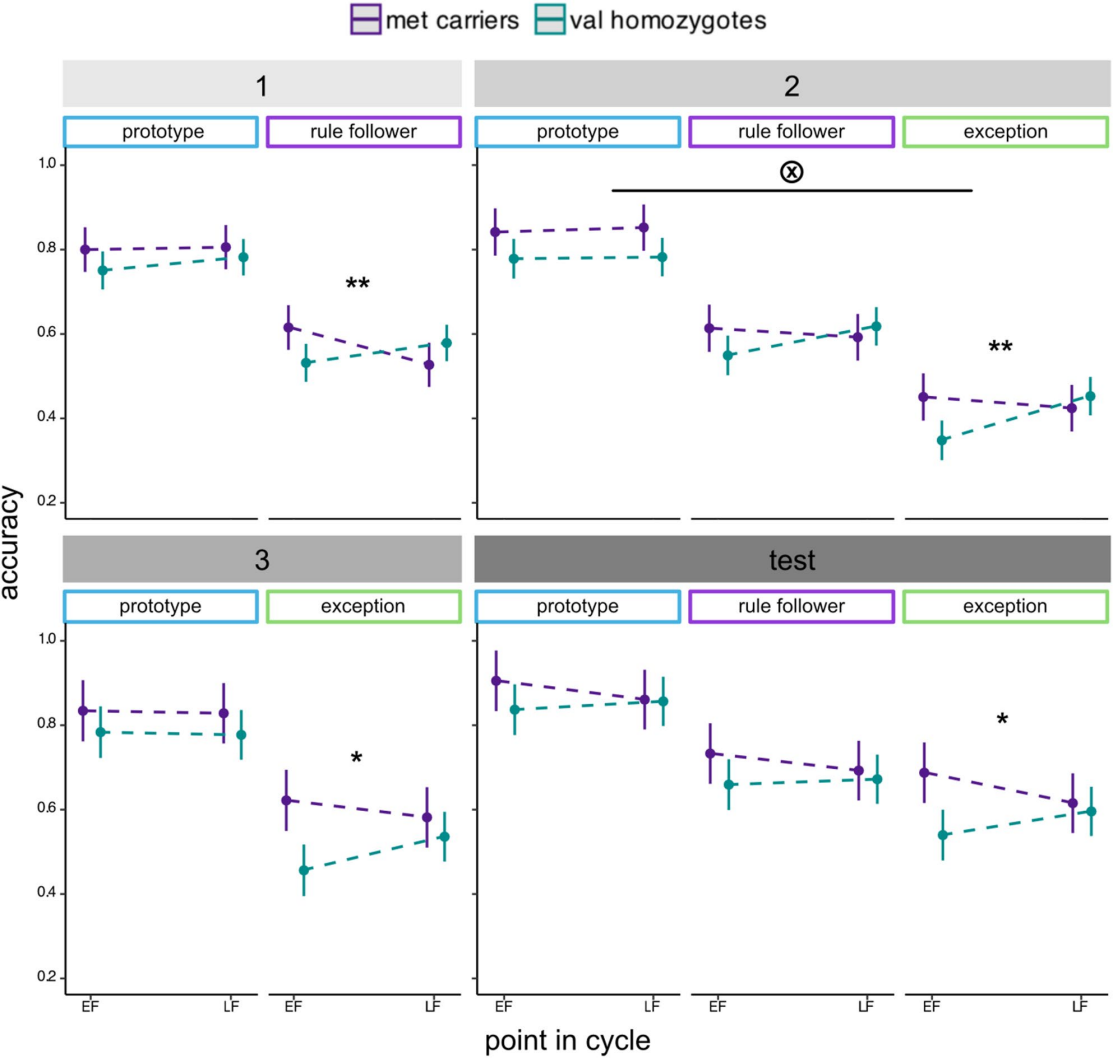
Effects of menstrual cycle session and BDNF Val66Met genotype (*met* carriers vs. *val* homozygotes) on rule-plus-exception category learning performance. The grey title bars indicate task blocks(1 = first learning block; 2 = second learning block; 3 = third learning block; test = test block). Participants were provided feedback on their categorization decisions during the three learning blocks while the test block contained no feedback, testing final learning of the category structure. Error bars denote 95% confidence intervals. “*” indicates *p* < 0.05, “**” indicates *p* < 0.01, and the tensor product symbol indicates a significant interaction between stimulus type, menstrual phase and genotype

Significant interactions between menstrual cycle phase and genotype revealed that *met* carriers had an advantage early on in learning – immediately after task commencement for rule-follower items (β = 0.14, SE = 0.04, t(170) = 3.05, *p* = 0.002; CI [0.050, 0.220]) and immediately after exception introduction for exception items (β = 0.13, SE = 0.05, t(280) = 2.73, *p* = 0.007; CI [0.038, 0.223]) – but only during the early follicular timepoint (Fig. 2). The same pattern remained significant for exception items during the third learning block (β = 0.12, SE = 0.06, t(168) = 2.16, *p* = 0.032; CI [0.013, 0.226]) and into the no-feedback test block (β = 0.13, SE = 0.06, t(280) = 2.11, *p* = 0.035; CI [0.011, 0.244]). The effects on prototype items were less notable, with a three-way interaction indicating a different pattern of BDNF/session interactions for prototype relative to exception items briefly emerging in the second learning block (β = −0.14, SE = 0.07, t(277) = −2.05, *p* = 0.041; CI [−0.266, −0.008]).

### Hormone-gene analysis

To better understand why *met* carriers outperformed *val* homozygotes in the early but not late follicular timepoint, we conducted follow-up hormone-gene interaction analyses. Due to a large individual variance in hormonal trajectories across the menstrual cycle, recent methodological guidelines suggest examining subsets of participants with ‘typical’ changes in hormonal levels across sampled points in the menstrual cycle [77]. Thus, we analyzed hormone-gene interactions in a subset of our participants who showed an increase in estradiol between the two sessions (*N* = 37). Due to a lack of standardized cycle phase hormone ranges and in order to avoid subjective decisions on magnitude of change, all participants showing an increase in estradiol were included.

**Fig. 3:**
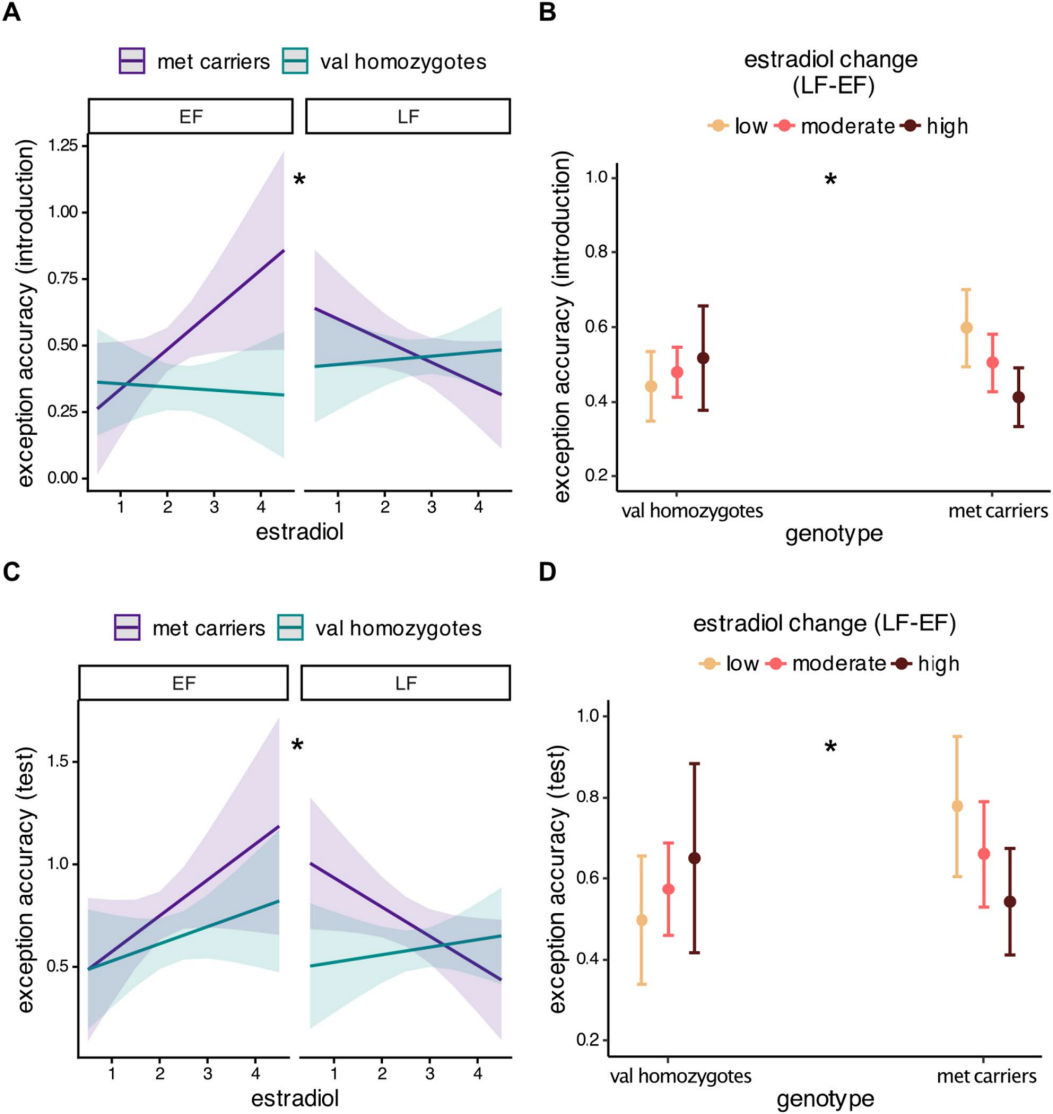
Estradiol effects on categorization accuracy for exceptions vary by genotype. Immediately after exception introduction, *met* carriers benefit from higher estradiol in the early follicular session but not in the late follicular session (**A**); *val* homozygotes’ performance increases and *met* carriers’ performance declines with higher increases in estradiol between sessions (**B**). Similar patterns are seen in the test block in terms of both absolute hormone level effects on *met* carriers and (**C**) the divergent effects of magnitude of change in estradiol levels for *val* homozygotes and *met* carriers (**D**). Error bars denote 95% confidence intervals. “*” indicates *p* < 0.05

As expected, participants’ estradiol levels were significantly higher in the late (M = 2.72, SD = 0.82) relative to early follicular (M = 2.10, SD = 0.70) timepoint (β = 0.61, SE = 0.08, t [36] = 7.43, *p* < 0.001; CI [0.452, 0.785]) while progesterone levels did not differ between timepoints (*p* > 0.05). Estradiol levels and their rate of change between sessions moderated the effect of genotype on categorization accuracy of exceptions. We found a significant interaction between session, genotype and estradiol levels immediately after exception introduction: *met* carriers benefitted from higher estradiol in the early but not in the late follicular timepoint (β = 0.26, SE = 0.11, t [60] = 2.42, *p* = 0.019; CI [0.066, 0.452]; Fig. 3A]. Additionally, there was a significant interaction between genotype and magnitude of change in estradiol levels between sessions, so that exception accuracy of *val* homozygotes in the late follicular timepoint increased with higher increases in estradiol, but the opposite was true for *met* carriers (β = 0.25, SE = 0.11, t [30] = 2.38, *p* = 0.024; CI [0.036, 0.471]; Fig. 3B].

Similar patterns emerged in the test block. There was a significant interaction between timepoint and estradiol in *met* carriers only, indicating that their performance increased with higher estradiol in the early follicular timepoint but decreased with higher estradiol in the late follicular timepoint (β = −0.32, SE = 0.12, t(39.2) = −2.67, *p* = 0.011; CI [−0.523, −0.070]; Fig. 3C]. There was also a significant interaction between genotype and magnitude of change in estradiol between sessions: performance of *val* homozygotes increased but performance of *met* carriers decreased with higher increases in estradiol (β = 0.39, SE = 0.18, t [30] = 2.18, *p* = 0.038; CI [0.024, 0.753]; Fig. 3D]. There were no significant hormone-gene effects on rule-follower categorization (all *p* > 0.05).

## Discussion

The current results provide the first evidence of an interaction between BDNF Val66Met genetic polymorphism and the human menstrual cycle in predicting cognitive performance, as well as the first investigation of BDNF Val66Met effects on category learning. *Met* carriers tend to outperform *val* homozygotes in the early but not late follicular point of the cycle, with the interaction potentially driven by *met* carriers' increased sensitivity to estradiol levels. Namely, while *met* carriers benefit from higher estradiol in the early follicular session when levels of estradiol are low, the opposite is true in the high-estradiol, late follicular session. In fact, the higher the increase in estradiol between the timepoints, the more *met* carriers’ performance decreases at the late timepoint.

These results are in line with prior work demonstrating menstrual cycle effects on category learning [62] and further advance the literature by directly implicating estradiol in category learning performance. Current results are convergent with findings suggesting that both BDNF and ovarian hormones have extensive neuromodulatory effects [23, 52, 78], including regulating dendritic spine density [79–82], synaptic plasticity [83–86], and cognitive function [1, 38]. Furthermore, BDNF is most abundantly expressed in the hippocampus [87, 88], a brain region rich with ovarian hormone receptors [89], highly sensitive to circulating levels of ovarian hormones [4], and critical for rule-plus-exception category learning [54, 55, 57].

While BDNF Val66Met polymorphism has been repeatedly implicated in hippocampal structure [31, 38], function [35, 37], and cognition [22, 38], results remain fairly mixed in terms of which allele combination confers advantage [17, 22]. While early work conceptualized the *met* allele as generally disadvantageous, it has since become evident that BDNF Val66Met effects on cognition are population- and domain-specific [39]. Our results not only provide the first evidence of BDNF Val66Met genotype impact on category learning but also suggest that some of the inconsistencies in the literature on BDNF Val66Met, learning and memory may be explained by individual differences in hormonal milieu.

Notably, BDNF expression is modulated by estradiol. BDNF expression closely follows endogenous ovarian hormone fluctuations across the menstrual cycle, with pre-ovulatory and mid-luteal peaks [42, 44], and similarly varies across the animal analogues of the menstrual cycle [30, 43, 45]. Estradiol also regulates BDNF expression in the hippocampus [41, 52, 90, 91], with BDNF mRNA in CA1 and CA3 fluctuating significantly across the estrous cycle in rodents [43]. In CA3, BDNF mRNA is higher in the high-estradiol phase in both *val* and *met* mice, but *met* mice have increased expression compared to *val* mice [45]. The distribution of the BDNF TrkB receptor also peaks in the high-estradiol phase of the estrous cycle [92] and TrkB mRNA in CA1 is higher in *met* mice. Similar hormone-genotype interactions may underlie the current results as both CA1 and CA3 are thought to be important for exception learning.

The fact that *met* carriers in the current study benefit from estradiol in the low- but not high-estradiol point in cycle suggests a potential dose-dependent effect of estradiol. These findings align with rodent work showing that while *met* mice have higher BDNF expression in CA3 in the presence of high estradiol, they nevertheless show worse cognitive performance [45]. Similarly, female *met* mice show better performance on the hippocampal spatial memory task in a low-estradiol condition [93]. Our findings are also in line with the only other study examining the interaction between the BDNF Val66Met genotype and hormone levels in predicting human cognition. Specifically, women homozygous for the *met* allele show inefficient hippocampal activation patterns in a pharmacologically manipulated high-estradiol condition [46].

In addition to the effects of overall estradiol levels, our *met* participants also show higher sensitivity to *changes* in estradiol levels. This aligns with the hormone sensitivity hypothesis [12, 13], which suggests that people vary in the extent to which they are behaviorally affected by changes in sex hormone levels. Extensive evidence, primarily from work on emotional functioning and affective disorders, suggests that typical changes in hormone levels across the menstrual cycle can have clinically significant effects on mood, cognition, and behaviour in specific subsets of the population [12, 13]. The current results provide preliminary evidence that this type of hormone sensitivity may partially vary by BDNF Val66Met genotype, which has itself been repeatedly implicated in psychiatric conditions [39, 94].

The significant interaction between point in cycle and BDNF genotype identified in the current work may help explain the variance in literatures on cognition across the menstrual cycle as well as BDNF Val66Met genotype and cognition. Due to the large variation in both menstrual cycle [2] and ovarian hormone [1, 3, 95] effects on cognition, it is necessary to account for interacting factors that may underlie individual differences. Similarly, it is evident from the highly mixed results on BDNF Val66Met effects on cognition [17, 22, 39] that it is not sufficient to examine genotype effects in isolation. Prior work has suggested that sex of participants is an important factor that may modulate the effects of BDNF genotype on cognition during the female reproductive lifespan [47, 48]. The current work extends this by demonstrating within-sex variability associated with the menstrual cycle.

Finally, the current results in a healthy younger population may have implications for research on BDNF Val66Met and mental processes in neuropsychiatric illnesses or in older individuals. They are of particular relevance to research on cognition in peri- and post-menopausal women, who are either experiencing or have experienced a drastic decline in estradiol levels, a hormonal transition known to affect cognition [96–100]. Furthermore, prior research suggests that BDNF Val66Met genotype has sex-specific effects in both healthy aging [101] and Alzheimer’s disease [102], such that the *met* allele confers risk in women but not in men, highlighting the importance of studying the interacting effects of sex, steroid hormones, and BDNF Val66Met both during and beyond the reproductive lifespan.

Major strengths of the current study include a well-powered sample, tight control of the testing timing in relation to days of the menstrual cycle including the use of multiple confirmation measures, and only recruiting participants who used period tracker apps or calendars so that key variables were not based on subjective measures such as counting [77]. However, future work would benefit from controlling for length of tracker app use for cycle length determination, as well as the use of multiple hormonal measures administered across the full length of the menstrual cycle, including the luteal phase which would enable an examination of the effects of progesterone at its peak. Multiple hormonal measures would also improve the capacity to determine exact menstrual cycle phase, which was limited in the current study, particularly as presence of anovulatory cycles, which are associated with overall lower ovarian hormone levels [103], could not be ruled out. Future work may also consider additional factors that may interact with BDNF and sex hormones to predict cognition, such as exercise [49, 104, 105] and affect [106–108]. Effects of serum BDNF levels, which fluctuate with estradiol across the menstrual cycle [42, 44] should also be considered. Finally, the extent of the interacting effects of the menstrual cycle and BDNF Val66Met genotype on cognition remains to be fully elucidated. While the RPE task used in the current study presents a very sensitive measure of hippocampal function [55, 109], and relies on hippocampal processes necessary for episodic memory such as rapid formation of conjunctive representations, and memory integration and differentiation [55, 57, 58, 110, 111] the generalizability of the current findings to other processes associated with episodic memory, as well as to other types of learning, needs to be evaluated in future studies.

## Conclusion

The current study bridges neuroendocrinology, cognitive psychology, and behavioral genetics to demonstrates an interactive effect of menstrual cycle phase and BDNF Val66Met on human cognition. It replicates and extends prior work on category learning across the menstrual cycle by directly implicating estradiol in category learning performance and identifying a significant source of individual variance modulating the impact of the menstrual cycle on categorization accuracy, while also highlighting a critical source of individual variance in the effects of BDNF Val66Met on cognition. The current results demonstrate the critical importance of considering interactions between biological systems when studying cognition.

## Supplementary Information

Supplementary Material 1

## Data Availability

Code, as well as data necessary to reproduce the main analyses will be made available on OSF upon publication: [https://osf.io/wfecv/](https:/osf.io/wfecv) Demographic data will not be shared in order to ensure that no sensitive genotype or hormone information can be linked to individual participants.
