# Supplementary material for: Menstrual cycle modulates the effect of BDNF Val66Met variant on category learning
Source: Biol Sex Differ. 2026 Mar 1;17:65. doi: 10.1186/s13293-026-00856-2 (PMC13059548; doi:10.1186/s13293-026-00856-2)
Supplement: Supplementary file 1 — Supplementary Material 1 [file 13293_2026_856_MOESM1_ESM.docx]

**Supplementary materials**

**Supplementary figure 1.** Recruitment flowchart


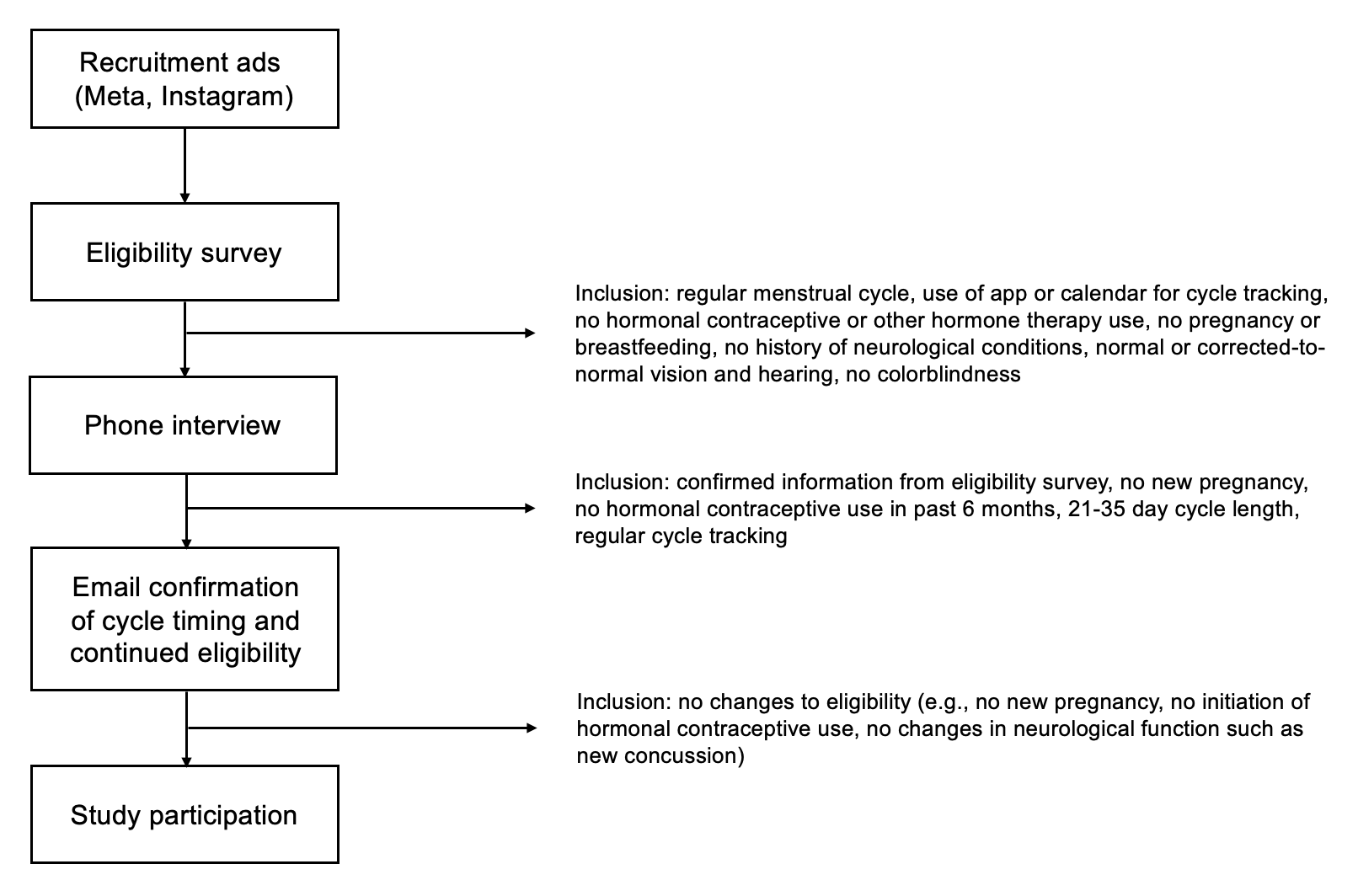


**Sensitivity analyses**

Sensitivity analyses including age and education as covariates yielded the same pattern of results as the original analyses. Significant interactions between menstrual cycle phase and genotype indicated that met carriers had an advantage early on in learning – immediately after task commencement for rule-follower items (β = 0.14, SE = 0.04, t(170) = 3.08, p = 0.002) and immediately after exception introduction for exception items (β = 0.13, SE = 0.05, t(277) = 2.73, p = 0.007) – but only during the early follicular timepoint. The same pattern remained significant for exception items during the third learning block (β = 0.12, SE = 0.06, t(168) = 2.16, p = 0.032) and into the no-feedback test block (β = 0.13, SE = 0.06, t(281) = 2.11, p = 0.036). The effects on prototype items were less notable, with a three-way interaction indicating a different pattern of BDNF/session interactions for prototype relative to exception items briefly emerging in the second learning block (β = -0.14, SE = 0.07, t(277) = -2.05, p = 0.041). Hormone analyses indicated a significant interaction between session, genotype and estradiol levels after exception introduction: met carriers benefitted from higher estradiol in the early but not in the late follicular timepoint (β = 0.23, SE = 0.08, t(58) = -2.59, p = 0.012), and a significant interaction between genotype and magnitude of change in estradiol levels between sessions, so that exception accuracy of val homozygotes in the late follicular timepoint increased with higher increases in estradiol, but the opposite was true for met carriers (β = 0.26, SE = 0.11, t(28) = 2.3, p = 0.029). Similar patterns emerged in the test block. There was a significant interaction between timepoint and estradiol in met carriers only, indicating that their performance increased with higher estradiol in the early follicular timepoint but decreased with higher estradiol in (the late follicular timepoint [β = -0.31, SE = 0.12, t(38.09) = -2.59, p = 0.014], and a marginally significant interaction between genotype and magnitude of change in estradiol between sessions so that performance of met carriers decreased with higher increases in estradiol (β = 0.39, SE = 0.19, t(28) = 2.04, p = 0.051). There were no significant hormone-gene effects on rule-follower categorization (all p > 0.05).

Sensitivity analyses controlling for reported medication use at time of testing (in n=6) yielded the same pattern of results as the original analyses. Significant interactions between menstrual cycle phase and genotype indicated that met carriers had an advantage early on in learning – immediately after task commencement for rule-follower items (β = 0.14, SE = 0.04, t(170) = 3.06, p = 0.003) and immediately after exception introduction for exception items (β = 0.13, SE = 0.05, t(280) = 2.73, p = 0.007) – but only during the early follicular timepoint. The same pattern remained significant for exception items during the third learning block (β = 0.12, SE = 0.06, t(168) = 2.16, p = 0.032) and into the no-feedback test block (β = 0.13, SE = 0.06, t(280) = 2.11, p = 0.036). The effects on prototype items were less notable, with a three-way interaction indicating a different pattern of BDNF/session interactions for prototype relative to exception items briefly emerging in the second learning block (β = -0.14, SE = 0.07, t(275) = -2.05, p = 0.041). Hormone analyses indicated a significant interaction between session, genotype and estradiol levels after exception introduction: met carriers benefitted from higher estradiol in the early but not in the late follicular timepoint (β = 0.26, SE = 0.1, t(59) = -2.42, p = 0.019), and a significant interaction between genotype and magnitude of change in estradiol levels between sessions, so that exception accuracy of val homozygotes in the late follicular timepoint increased with higher increases in estradiol, but the opposite was true for met carriers (β = 0.26, SE = 0.11, t(29) = 2.45, p = 0.021). Similar patterns emerged in the test block. There was a significant interaction between timepoint and estradiol in met carriers only, indicating that their performance increased with higher estradiol in the early follicular timepoint but decreased with higher estradiol in (the late follicular timepoint [β = -0.31, SE = 0.12, t(37.99) = -2.61, p = 0.013], and a significant interaction between genotype and magnitude of change in estradiol between sessions so that performance of met carriers decreased with higher increases in estradiol (β = 0.39, SE = 0.18, t(30) = 2.16, p = 0.037). There were no significant hormone-gene effects on rule-follower categorization (all p > 0.05).
